# Novel Anti Double-Stranded Nucleic Acids Full-Length Recombinant Camelid Heavy-Chain Antibody for the Detection of miRNA

**DOI:** 10.3390/ijms23116275

**Published:** 2022-06-03

**Authors:** Malgorzata Czarnecka, Ulrike Weichelt, Stefan Rödiger, Katja Hanack

**Affiliations:** 1 Institute of Biochemistry and Biology, University of Potsdam, Karl-Liebknecht-Str. 24-25, 14476 Potsdam, Germany; maczarne@uni-potsdam.de (M.C.); weichelt@uni-potsdam.de (U.W.); 2 Faculty of Health Sciences Brandenburg, Brandenburg University of Technology, Cottbus-Senftenberg, Universitätsplatz 1, 01968 Senftenberg, Germany; stefan.roediger@b-tu.de

**Keywords:** antibody, camelid antibody, heavy-chain-only antibody, miRNA, nucleic acids, novel biomarkers

## Abstract

The discovery that certain diseases have specific miRNA signatures which correspond to disease progression opens a new biomarker category. The detection of these small non-coding RNAs is performed routinely using body fluids or tissues with real-time PCR, next-generation sequencing, or amplification-based miRNA assays. Antibody-based detection systems allow an easy onset handling compared to PCR or sequencing and can be considered as alternative methods to support miRNA diagnostic in the future. In this study, we describe the generation of a camelid heavy-chain-only antibody specifically recognizing miRNAs to establish an antibody-based detection method. The generation of nucleic acid-specific binders is a challenge. We selected camelid binders via phage display, expressed them as VHH as well as full-length antibodies, and characterized the binding to several miRNAs from a signature specific for dilated cardiomyopathy. The described workflow can be used to create miRNA-specific binders and establish antibody-based detection methods to provide an additional way to analyze disease-specific miRNA signatures.

## 1. Introduction

Micro ribonucleic acids (miRNAs) are small (17–25 nucleotides) non-coding RNA, that play an essential role in regulating post-transcriptionally gene expression. As a part of the RNA-induced silencing complex (RISC), they bind complementary imperfect mRNA sequences thus modulating or silencing the activity of their mRNA targets [1]. Altered miRNA profiles have been discovered in multiple tissues and body fluids, that have been associated with the onset, progress, and prognosis of several serious diseases such as cancer, neurological disorders, and cardiovascular and myocardial diseases [2,3,4,5,6,7,8,9]. In association with inflammatory and virally induced cardiomyopathies and dilated cardiomyopathy (DCM), the miRNAs *homo sapiens* (hsa)-let-7f-5p, hsa-miR-30a-3p, hsa-miR-93-5p, hsa-miR-197-3p, hsa-miR-223, and hsa-miR-379-5p showed an altered expression profile [7,10]. There is rising interest in elucidating miRNA expression patterns and their functions because they represent promising second-generation biomarkers for new diagnostic approaches under physiological and pathophysiological conditions. We took it as an opportunity to develop and establish a phage display protocol for the selection of anti-nucleic acid binders using the altered miRNA expression profile of DCM.

The generation of nucleic acid-specific antibodies is a high challenge, especially with regard to specificity and cross-reactivity. In certain autoimmune diseases such as systemic lupus erythematosus (SLE) specific immunoglobulins against double-stranded DNA (ds DNA) are generated in vivo and used as specific biomarkers in the diagnostics of such disorders [11,12,13,14]. This implies that the human immune system is able to address this challenge. Antibodies from autoimmune patients and autoimmune disease-related animal models have been successfully isolated and engineered for use as diagnostic and research tools.

In the last century, there have been several approaches to generate antibodies against DNA, alpha oligonucleotides, DNA:RNA hybrids, virus RNA, nucleotides, and RNA among others by hybridoma technology [15,16,17,18,19]. Hu et al. summarized several studies in which anti-nucleic acid antibodies were generated and proposed their possible use in clinical and or genomic detection and diagnostics [20]. The experimental in vivo generation has been proven to be very challenging or unsuccessful since native DNA and RNAs are poor antigens that will be tolerated or degraded by the animal host reaction. To induce measurable immune reactions, it is recommended to use nucleic acids complexed with carrier proteins or synthetic peptides, chemically modified ribonucleotides, or high molecular weight polynucleotides in general [21,22,23]. Further, it is difficult to elicit antibodies having a high affinity to each type of nucleic acid without showing cross-reactivity with others. The anti-DNA:RNA hybrid antibody based on the one generated by Nakazato in the 1970s against synthetic φX174 DNA:RNA hybrid [17] is one of the few antibodies that made it to a (commercially available) customized product, that can be purchased via various companies. This antibody was proven to bind DNA:RNA hybrids and poly(I)-poly(dC) equally but not single-stranded DNA, ds DNA, or RNA [24].

In recent years, the variable domains of camelid heavy-chain-only antibodies have become more important for their possible application in the diagnostic due to their advantages [25]. The variable domains of camelid heavy-chain-only antibodies (VHHs or nanobodies) serve as the smallest known antigen-binding domains with a molecular weight of only 12–15 kDa derived from naturally occurring antibodies. Further, they possess a very high thermal resistance and physicochemical stability resulting from the decreased hydrophobicity and are stable at high pH values, high alcohol concentration, and chaotropic agents [26,27,28]. The VHH domain is composed of four frameworks and three domains referred to as complementarity determining regions (CDRs) instead of six as in the variable domains of heavy and light chain in a conventional antibody [29]. Within the framework 2 the highly conserved amino acids Val37, Gly44, Leu45, and Trp47 are substituted by smaller and/or hydrophilic amino acids such as Phe or Tyr37, Glu44, Arg or Cys45, and Gly or Ser, Leu, Phe47 (the position of the amino acids are numbered according to the Kabat numbering system) [30]. These four amino acid substitutions are referred to as hallmarks and are used to identify the antigen-binding domain as VHH. The hydrophobic amino acids (Val37, Gly44, Leu45, Trp47) assure the linkage between VH and VL in conventional antibodies.

**Figure 1:**
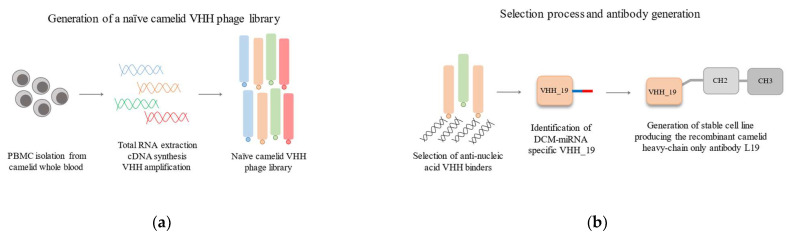
Schematic overview of performed phage display procedure to generate anti-short nucleic acid camelid binders. (**a**) The first step was the generation of a naïve camelid VHH phage library. The peripheral blood mononuclear cells (PBMCs) were isolated from the camelid whole blood. Next, the total RNA was extracted. cDNA synthesis and VHH amplification were performed to build a naïve camelid VHH phage library. (**b**) After several panning rounds, the potential anti-DCM-miRNA VHH_19 binder was identified. In order to produce the recombinant full-length camelid antibody, the DNA sequence was expanded by the introduction of the Fc region of camelid IgG2b instead of His (blue bar) and HA (red bar) tags.

To address the point of establishing a workflow for the generation of nucleic acid-specific antibodies and using them for diagnostic assay systems, we created a camelid-naïve cDNA library encoding diverse VHHs. VHH fragments against nucleic acids were selected by several panning rounds. The miRNA-223 which belongs to the altered miRNA expression profile of DCM was chosen as a model miRNA as antigen. By analyzing potential miRNA binders, we identified one clone (VHH_19) that showed a specific response to ds miRNA. By introducing the camelid IgG2b constant region to the VHH_19 we generated the recombinant full-length heavy-chain antibody L19 (Figure 1), which was expressed in HEK-293 cells. The purified L19 antibody was tested in a direct ELISA showing the binding specificity for ds miRNA. Our results demonstrate that anti-ds miRNA antibodies may contribute as useful tools for detecting and analyzing miRNA.

## 2. Results

### 2.1. Selection of ds miRNA VHH Binder

**Figure 2:**
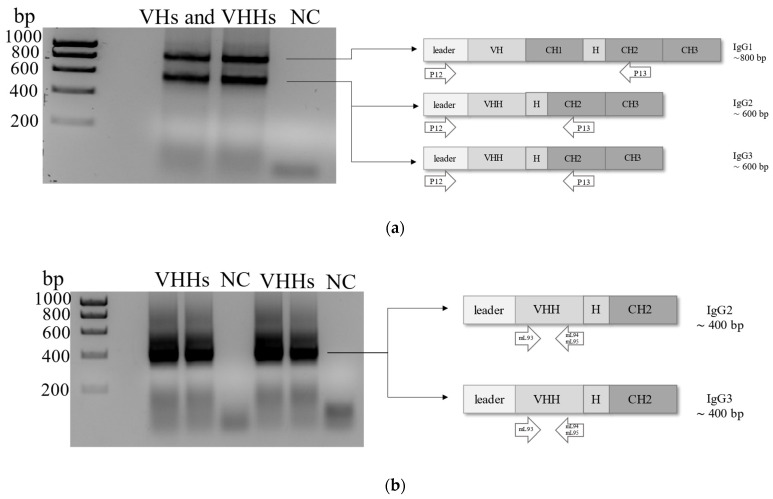
Agarose gel (1.5%) electrophoresis after the amplification of camelid VHs and VHHs. (**a**) The upper band at about 800 bp corresponds to the leader-VH-hinge-CH1-CH2 sequence of camelid conventional antibodies (IgG1) and the lower band at about 600 bp to the leader-VHH-hinge-CH2 sequence of unique heavy-chain-only antibodies. Amplicons of 600 bp were used as templates for the nested PCR. (**b**) Nested PCR amplified the VHH sequences of about 400 bp only (without leader and hinge region) using the forward primer mL93 and two reverse primers mL94 and mL95. For each PCR, DEPC-treated water was included as a negative control (NC, no template control). Hyperladder I™ 1 kb was used as DNA molecular weight marker (Bioline, London, Great Britain).

The total RNA isolated from naïve camelid PBMCs was transcribed into cDNA. The first PCR amplified the region between the leader sequence and the CH2 domain of both VH and VHH genes of conventional and heavy-chain antibodies (non-conventional antibodies) resulting in two bands with the sizes of about 800 bp and about 600 bp (Figure 2a). The 600 bp band was used as a template for the nested PCR to amplify the camelid VHH gene repertoire providing a 400 bp band (Figure 2b). The amplified VHH gene repertoire was cloned into the digested phagemid pCOMB3x to obtain a naïve VHH phage library with 6.2 × 10^6^ members. 

**Figure 3:**
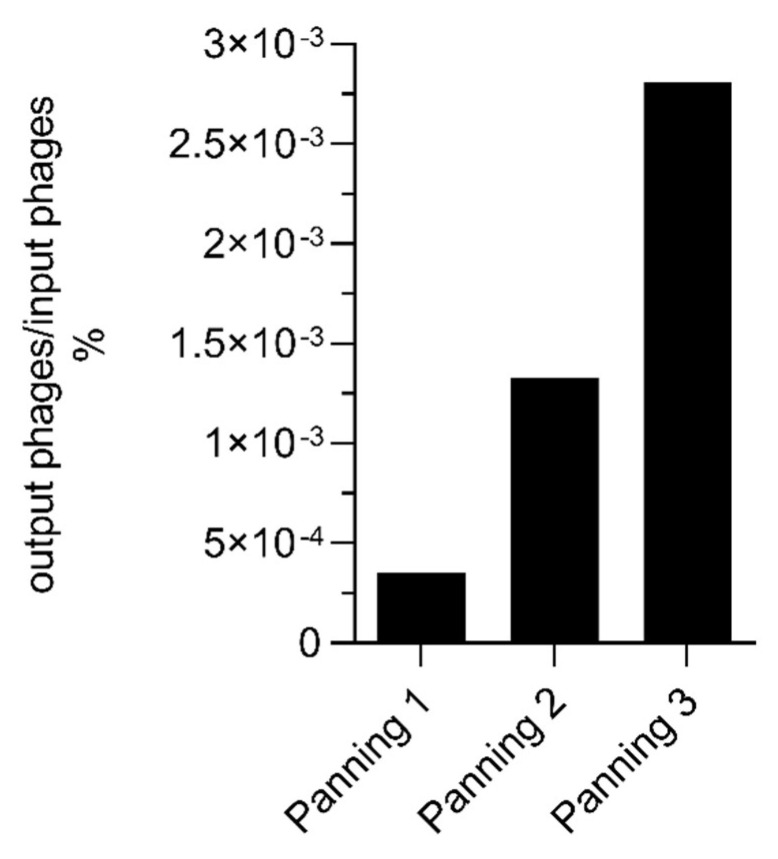
Percentage enrichment of polyclonal phage particles displaying different VHH fragments on their surfaces. The percentage enrichment was calculated by the division of the titer of output phages and titer of input phages multiplied by 100% for each panning round.

The naïve phage library was panned over three rounds for anti-miRNA VHHs. The polyclonal phage particles were pre-incubated with the carrier protein avidin and the unbound particles were then incubated with the synthetic model ds miRNA-223. The titer of the eluted phage particles increased during the three panning rounds indicating the enrichment of phage particles (Figure 3).

**Figure 4:**
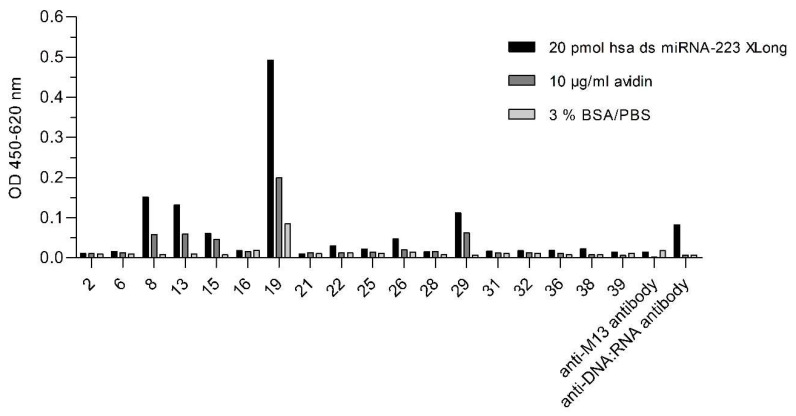
Monoclonal phage ELISA for the detection of hsa ds miRNA-223 XLong-specific VHH binders. Eighteen clones were selected from the last round of panning to produce the monoclonal phage particles. Their binding preference toward the hsa ds miRNA-223 XLong (20 pmol), a carrier protein (10 µg/mL), and blocking solution (3% BSA/PBS) was investigated. Bound monoclonal phage particles were detected by the secondary antibody anti-M13_HRP_ (1:7000). The binding of anti-M13_HRP_ to the antigens only was also performed. Additionally, a coating control for the immobilized hsa ds miRNA-223 XLong conjugated on avidin was carried out using the commercially available anti-DNA:RNA antibody (clone S9.6) which was detected with goat anti-mouse IgG_HRP_ (Fcγ).

After the last panning round, 18 clones were chosen for the generation of monoclonal phage particles each displaying only one type of VHH fragment. The monoclonal phage particles were tested for the binding to the hsa biotinylated (bio) double-stranded (ds) miRNA-223 with a five times repetitive extended sequence (XLong) conjugated to avidin and additional for cross-reaction against the carrier protein and the blocking solution. Moreover, the use of anti-DNA:RNA antibody (S9.6) instead of VHH displaying phage particles represented the coating control for the miRNA. Of these 18 clones, clone number 19 yielded the highest ELISA signals for ds miRNA (Figure 4) and was chosen for the soluble expression of VHH referred to as VHH_19.

### 2.2. Expression and Purification of Recombinant VHH_19

**Figure 5:**
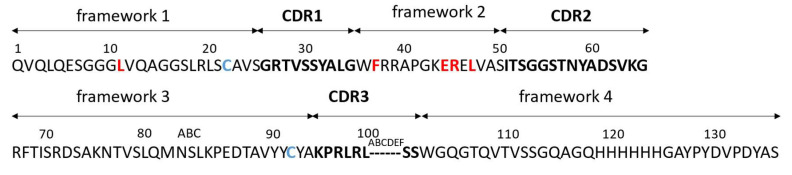
The amino acid sequence of VHH_19 displays the three CDRs (black, bold) and four frameworks. The residues were numbered according to the Kabat numbering scheme. The four hallmark amino acid changes at positions 37, 44, 45, and 47 within framework 2 are marked in red. Moreover, the sequence showed the two conserved Cys residues at positions 22 and 92 (blue). The His- and HA-tags were located at the C-terminus.

**Figure 6:**
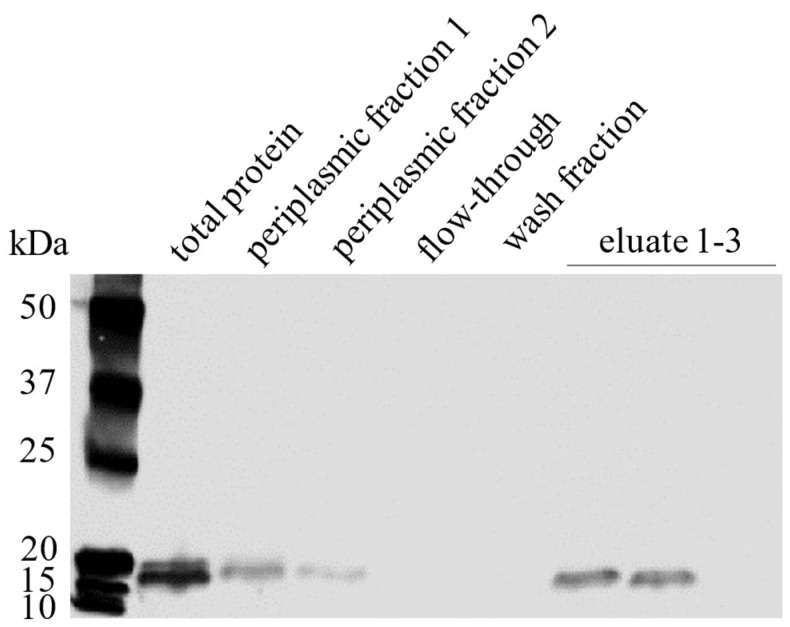
Western blot of expressed VHH_19 and its presence in purification steps. Following samples, total protein, periplasmic fraction 1 and 2, flow–through, wash fraction, and eluate 1–3 were collected after the expression and during the Ni–NTA purification process. Protein samples were separated on a 12% TGX gel (BioRad) by SDS–PAGE and transferred to a nitrocellulose membrane by semi–dry WB. The detection of VHH_19 was performed using a primary anti–HA–tag antibody diluted 1:5000 in TBS–T/5% goat serum. The primary antibody was detected by an HRP-conjugated goat anti-mouse IgG Fcɣ antibody diluted 1:10,000 in TBS–T/5% goat serum. Precision Plus Protein TM WesternCTM Standard (BioRad, München, Germany) was used as a protein standard molecular weight marker (first lane).

From the positive clone 19, the plasmid pCOMB3x-VHH_19 was isolated and sequenced. The amino acid sequence of VHH_19 (Figure 5) was numbered according to the Kabat numbering system. The four typical aa substitutions were detected at positions 37, 44, 45, and 47. Moreover, the sequence possesses two conserved Cys residues at positions 22 and 9, which are typical for VHHs. The sequences for His-tag (for purification) and HA-tag (for detection) are present after framework 4. VHH_19 clone was expressed in a soluble form in the periplasmic space of *E. coli* HB2151 and purified via Ni-NTA affinity chromatography. The presence of VHH_19 in the elution fractions was confirmed by Western blot (WB). The protein bands (Figure 6) between 15 and 20 kDa were detected by an anti-HA-tag antibody in the following collected samples: total protein, periplasmic fraction 1 and 2 (before and after the dialysis with native binding buffer (NBB) containing 10 mM imidazole) and in the elution fractions 1 and 2 indicating the expressed VHH_19. No protein bands could be detected in the flow-through or in the wash fraction. The VHH_19 concentration of 2284 µg/mL was determined via the bicinchoninic acid (BCA) assay.

### 2.3. Generation and Purification of Recombinant Full-Length Camelid Heavy-Chain Only Antibody L19

**Figure 7:**
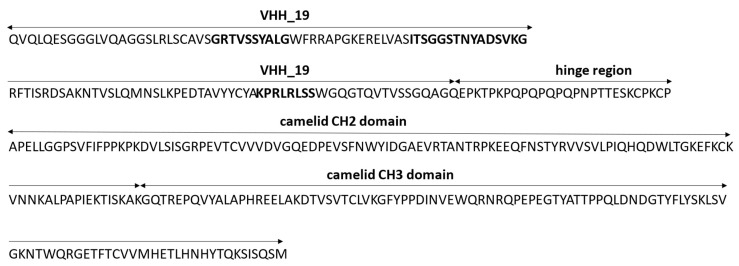
The amino acid sequence of the camelid recombinant full-length L19 antibody. The CDRs are highlighted in bold.

**Figure 8:**
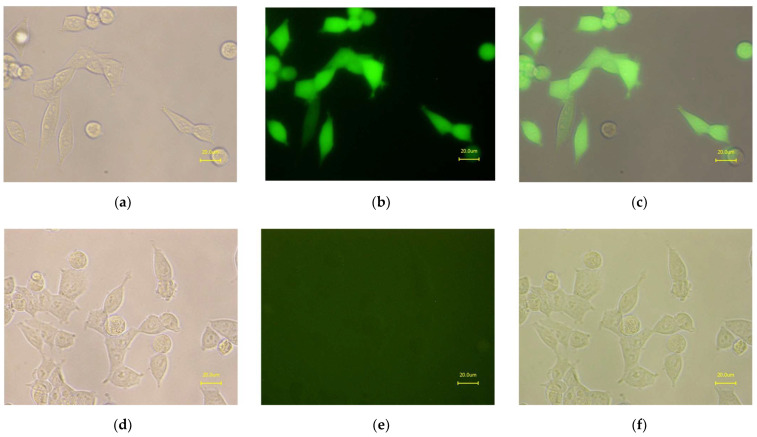
Presented are HEK-293 cells producing L19 antibody (stable cell line) after puromycin-induced selection in the bright-field (**a**), fluorescence (**b**), and overlay (**c**). In comparison, the HEK-293 wild-type cells are presented in the bright-field (**d**), fluorescence (**e**), and overlay (**f**) showing no GFP signal. The pictures were taken with the fluorescence microscope BZ-81000 (Keyence, Japan) with the object lens Plan Fluor ELWD 20×/0.45 (Ph1 DM ∞/0-2 WD 7.4, Nikon, Japan) under optical magnification 40×.

**Figure 9:**
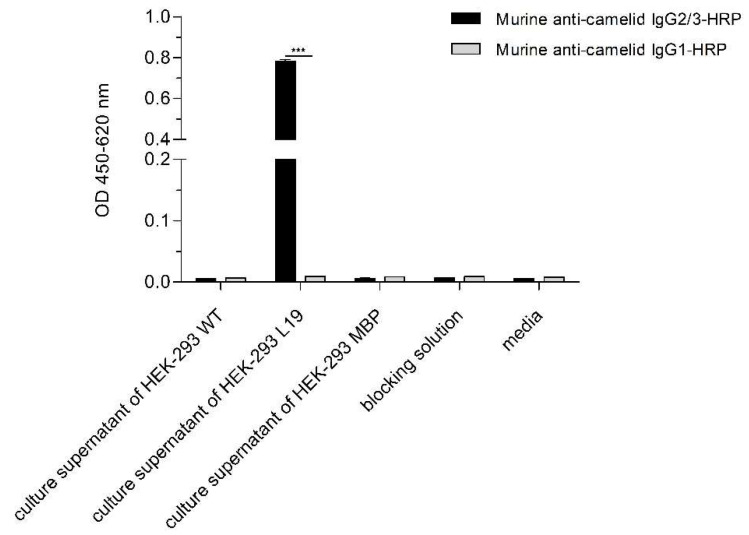
The culture supernatant of transfected HEK-293 cells was checked for the production of secreted L19. A murine anti-camelid IgG1/2/3 antibody (5 µg/mL) was coated onto the solid phase. Bound L19 antibodies were detected by the secondary HRP-labeled murine anti-camelid IgG2/3 and IgG1 antibodies (1:1000) discriminating between camelid IgG subclasses. The statistical significances between the different groups detected by two secondary antibodies were determined by an unpaired *t*-test with *** *p* < 0.001 (n = 3).

In the microbeads-based pre-experiment, it was shown that VHH_19 binder was able to recognize nucleic acids in a concentration-dependent manner and to differentiate between healthy and diseased samples (Appendix A). In order to provide a camelid heavy-chain-only full-length antibody format for further investigations, an appropriate vector pMC19 encoding the Fc region of camelid IgG2b antibody was designed for the mammalian expression and transfected into HEK-293 cells. The amino acid sequence of the full-length recombinant L19 antibody is presented in Figure 7 showing that the L19 antibody is composed of the same antigen-binding domain as VHH_19 and the camelid Fc tag. The Fc tag was used to employ another purification (protein A chromatography) and detection strategy. Due to the co-expression of L19 and GFP, the transfection efficiency was verified with fluorescence microscopy. Thus, all the cells detected with GFP expression secrete L19 into the culture medium. The puromycin-induced selection was introduced to select stable pMC19-transfected cell clones (Figure 8c). In comparison, the HEK-293 wild-type (WT) cells showed no GFP signal meaning that there is no antibody production. This fact was also proven with an ELISA (Figure 9).

The secretion of L19 into the culture media was investigated with an ELISA (Figure 9). As expected, L19 could be detected by a secondary murine anti-camelid IgG2/3-HRP antibody providing an optical density of 0.8. To demonstrate that L19 is a heavy-chain-only antibody, an HRP-labeled murine monoclonal anti-camelid IgG1 antibody was used in ELISA showing a significantly weaker signal (Figure 9) indicating that L19 is of an IgG2/3 isotype. In addition, the used set of negative controls (media with supplements, culture supernatant of HEK-293 WT cells, culture supernatant of HEK-293 cells producing a membrane-bound protein (MBP), and blocking solution) yielded very low signals. From 500 mL collected culture supernatants, we were able to purify 7.5 mg of L19 antibody.

### 2.4. Detection of L19 with Western Blot Analysis

**Figure 10:**
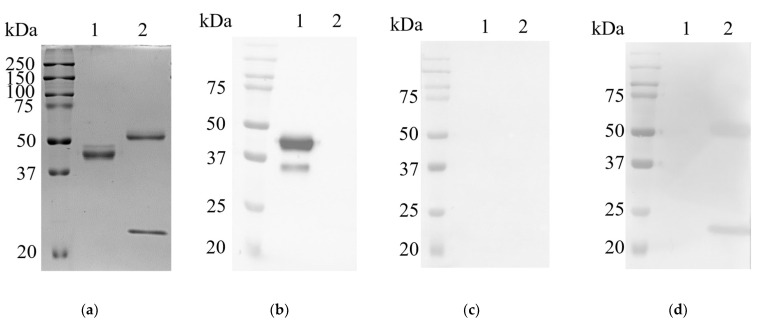
SDS-PAGE and Western blot analysis of camelid heavy-chain-only antibody L19 and a conventional murine monoclonal IgG1 antibody in contrast. The separated antibodies were transferred to a nitrocellulose membrane under semi-dry conditions. Precision Plus Protein™ Dual Color Standard, (BioRad, München, Germany) was used as protein standard molecular weight marker (**a**) both antibodies (each 2 µg) were separated on 10% polyacrylamide gels under reducing conditions and stained by Coomassie staining solution. (**b**) Detection of protein bands performed with HRP-conjugated murine anti-camelid IgG2/3 antibody. (**c**) Detection performed with HRP-conjugated murine anti-camelid conventional antibodies IgG1. (**d**) Detection of conventional murine antibody performed with HRP-conjugated goat anti-mouse IgG (H + L).

The recombinant camelid L19 and a murine monoclonal antibody were analyzed via SDS-PAGE and Western blot under reducing conditions (Figure 10). The SDS-PAGE result showed a prominent protein band at 40 kDa (lane 1, Figure 10a) indicating the camelid hinge, CH2, and CH3 region plus the VHH_19 domain of the full-length L19. Moreover, there is a weaker band over the 40 kDa band which may represent a glycosylated version of L19. Additional sequence analysis revealed no glycosylation sites within the VHH_19 but in the sequence for the Fc part of the camelid IgG2b, one glycosylation site at amino acid Asn177 could be identified. The glycosylation in the Fc part did not influence the antigen-binding of L19 but led to the weaker band above 40 kDa as shown in Figure 10a. The murine monoclonal antibody was used to visualize the structural difference between the conventional antibody consisting of a heavy (55 kDa) and a light chain (25 kDa) and the heavy-chain-only antibody (lanes 1 and 2, Figure 10a).

The L19 antibody could be detected with an HRP-labeled anti-camelid IgG2/3 antibody providing the band at 40 kDa (lane 1, Figure 10b). Due to the molecular weight of VHH_19 of approximately 15 kDa, the weaker band under 37 kDa showed only the camelid Fc region of L19. The heavy and light chain of the murine antibody was detected with goat anti-mouse IgG (H + L) antibody_HRP_ (lane 2, Figure 10d). As expected, there were no protein bands of L19 and murine antibody detected with the HRP-conjugated anti-camelid IgG1 antibody (Figure 10c).

### 2.5. ELISA with the Purified L19

**Figure 11:**
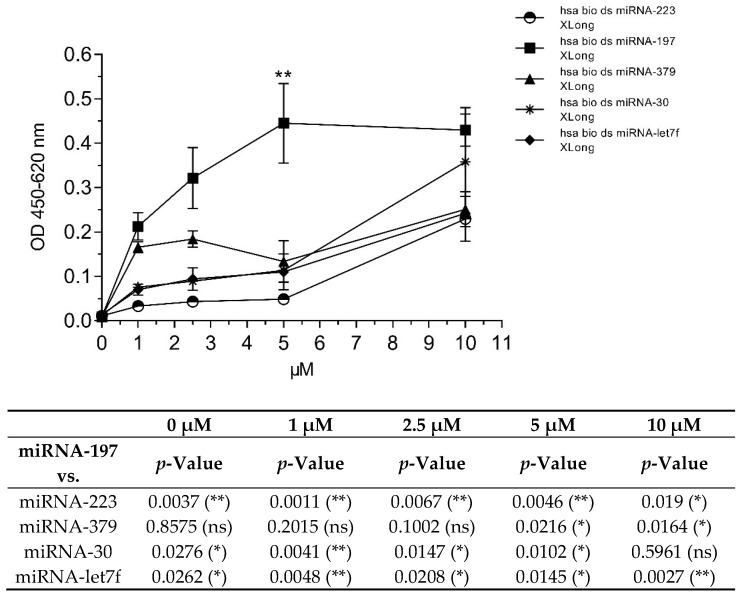
ELISA for the investigation of binding specificity to different repetitive extended hsa ds miRNAs conjugated with biotin. L19 (25 µg/mL) was coated onto the solid phase and incubated with five concentrations (0, 1, 2.5, 5, and 10 µM) of miRNAs (hsa bio ds miRNA-223, -197, -379, -30, and -let7f XLong. The concentration of 0 µM miRNA served as the negative control for the proof that neither the blocking solution nor the detection reagent SAV-HRP was bound by L19. The detection of bound miRNA was performed with HRP-labeled SAV. The statistical significances between the concentrations compared to miRNA-197 were determined by an unpaired *t*-test with ns *p* > 0.05, * *p* ≤ 0.05, ** *p* ≤ 0.01 (n = 4).

The recombinant full-length camelid antibody L19 was tested via ELISA for the binding specificity to hsa bio ds miRNA-233, -197, -379, -30, and let7f XLong which belong to the altered expression profile of DCM (Figure 11). L19 was coated onto the solid phase of an ELISA plate and incubated with different concentrations of biotinylated ds miRNAs. The bound miRNAs were detected by the HRP-labeled streptavidin (SAV) binding to the biotinylated part of miRNAs. According to the results in Figure 11, L19 possessed the highest binding preference for the hsa bio ds miRNA-197 XLong. L19 recognized hsa bio ds miRNA-379, -30, and -let7f XLong with moderate affinity. Moreover, hsa bio ds miRNA-223 XLong was detected with significantly reduced binding specificity within the DCM miRNA panel.

## 3. Discussion

Due to the rising interest in miRNA as novel biomarkers, we used the altered miRNA expression profile of DCM to generate an anti-nucleic acid binder. The common methods for the measurement of miRNA and their profile rely on nucleic acid probes-based techniques such as quantitative real-time PCR (qPCR), next-generation sequencing (NGS), and multiplex miRNA profiling assays or MicroRNA arrays [31,32,33]. These techniques display a high sensitivity but may differ in specificity and are time-consuming procedures. For example, qPCR analyses require careful sample preparation and the conversion of miRNA into cDNA. Because of the shortness of miRNA, it is necessary to incorporate additional stem-loop structures or poly-adenosine nucleotides before the actual qPCR can be driven. In contrast to the antibody-based detection method, their use is limited for routine diagnostics due to their complexity. Antibody-based detection methods are performed worldwide as standard applications in laboratory medicine. Immunoassays are less prone to interferences or contaminations. Their performance does not require expensive reagents, equipment tools or specialized personnel. Especially for the miRNA measurement, there is also no need to convert the miRNA into cDNA first. With the described workflow for the generation of miRNA-specific binders, we want to provide an additional way for the detection of miRNA.

As mentioned before, the development of anti-nucleic acid antibodies by the common hybridoma technology is challenging due to the low immunogenicity of nucleic acids. The data presented in this study may give new insights into the suitability of camelid phage display technology for the generation of VHHs against miRNAs. Here, we showed the identification of miRNA binders from the generated naive VHH phage library with 6.2 × 10^6^ members. The constructed VHH library is noticeably smaller than the reported ones in the literature (up to 10^11^ members) [34]. Nevertheless, it was described that antigen-binding domains such as VHHs could be recovered also from relatively small naïve phage libraries [35].

We selected the model antigen ds miRNA-223 from the altered miRNA expression profile of DCM. After several panning rounds against the ds miRNA-223, we have found a potential anti-nucleic acid binder referred to as VHH_19. In the first pre-experiments, VHH_19 was immobilized onto microbeads and the recognition of 0.1 fmol/µL miRNA-93 was detected (Appendix A). Moreover, the first tests with miRNA isolated from the serum of healthy and diseased donors were performed. The distinction in the recognition between the miRNA of healthy and diseased donors by the immobilized VHH_19 could be shown (Appendix A). However, the small size of VHH (12–15 kDa) and a decreased probe accessibility can be disadvantageous, especially when used for in vitro diagnostic systems [36]. To improve the probe accessibility, VHHs can be modified by introducing peptides at the C-terminus or by the fusion to the Fc region [36,37]. With the introduction of the Fc region, higher signals can be achieved and other assay formats can be performed. Thus, we decided to expand the identified VHH_19 with camelid Fc of IgG2b to generate a full-length camelid recombinant antibody L19 expressed in a mammalian expression system.

We were able to perform the ELISA test with a full-length L19 antibody to verify the anti- nucleic acid binding specificity. The highest signal was achieved for ds miRNA-197 at 5 µM. In comparison, the signals for the other ds extended miRNA detected by L19 were weaker. The sequence homology between the single miRNAs within the DCM profile is not high and there is no clear pattern or base pair motif that could be linked to the preference for miRNA-197. One point that might explain the phenomenon is the formation of loops within the miRNA sequences during hybridization. Two single RNA strands in directions 5′-3′ and 3′-5′ were synthesized and hybridized to generate the double-stranded miRNA used for the direct ELISA. The perfectly matched double-stranded miRNA sequence is not guaranteed during the hybridization step. The formation of loops within the sequence may occur with a high probability of causing different miRNA structures. There is no possibility to predict the miRNA structure with ds miRNA because the available bioinformatic structure prediction tools use single-stranded RNA sequences as input. The batch of ds miRNA-197 used for the direct ELISA could have a high degree of imperfect hybridization meaning the presence of inner loops in comparison to the other miRNAs. There is no possibility to measure or analyze the degree of hybridization as a melting curve analysis would not provide reliable results for such short ds miRNAs.

The ds miRNA-93 and the ds miRNA-223 bound by VHH_19 in the preliminary experiment (Table A1 and Table A2) and the ds miRNA-197 bound by L19 showed a higher homology degree between each other than with other miRNAs in the DCM miRNA panel (alignment not shown). Moreover, the ds miRNA-223 and the ds miRNA-197 were described to be implicated in endovascular inflammation and platelet activation and can be used as biomarkers to diagnose coronary artery disease [38]. The novel recombinant camelid antibody L19 can, therefore, be a useful tool for the further establishment of antibody-based miRNA detection. Recently, a new study was published that describes the antibody-based miRNA detection on the basis of the multiplex microchamber diffusion assay using the anti-hybrid DNA:RNA antibody (clone S9.6) [39]. These results in combination with the novel generated camelid miRNA binder may contribute to the further establishment or to the development of novel strategies for miRNA detection in a sequence-specific manner. The anti-hybrid DNA:RNA antibody (S9.6) is the most commonly used antibody for the development of antibody-based assays for the detection of miRNA [40,41]. In 2008, Ye and colleagues developed synthetic antibodies by using the phage display method to recognize structured RNA [42]. Recently, a Fab fragment was generated from a naïve antigen-binding fragment combinatorial phage library against the brain cytoplasmic 200 (BC200) RNA [43]. The isolated Fab is believed to recognize a domain of BC200 RNA in a sequence-dependent and conformation-based manner. However, these recombinant antibody formats are designed to recognize large and structured nucleic acid sequences.

We have demonstrated that by using the well-established phage display technology it is possible to generate recombinant antibodies against very short nucleic acid sequences which can be used to establish novel strategies for miRNA detection.

## 4. Materials and Methods

### 4.1. Used miRNAs

For the purpose of this study the miRNAs of the altered miRNA expression profile in association with DCM were purchased from Riboxx GmbH (Dresden, Germany). The sequence of each miRNA was repetitively extended five times and biotin was conjugated at the 5′end of the sense strand. The synthetic generated miRNAs were double stranded.

### 4.2. Extraction of Total RNA from Camelid Peripheral Blood Mononuclear Cells

Peripheral blood mononuclear cells (PBMCs) were isolated from fresh whole blood from three different naïve camelids (llama, alpaca, and huarizo) according to [44,45]. Finally, pellets were homogenized in 5 mL RNAPure^TM^ peqGOLD (VWR, Dresden, Germany) and stored at −80 °C for further treatments. Total RNA isolation was performed by the conventional phenol–chloroform extraction following the manufacturer’s instructions. Phase lock gel tubes (PLG, Quantabio) were used to yield higher RNA amounts. Finally, RNA concentrations were measured by UV spectroscopy at 260 and 280 nm.

### 4.3. Construction of Naïve Phage Library

CopyDNA (cDNA) was synthesized from 1 µg total RNA using the RevertAid First Strand cDNA Synthesis Kit (Thermo Fisher Scientific, Waltham, MA, USA). VHH sequences were amplified by two-step PCR using the primers listed in Table 1. The amplification of the camelid VHH repertoire was performed as previously described [44,46,47]. PCR products with a size of 400 bp were used for the construction of the naïve phage library. After the purification from the 1.5% agarose gel and digestion with SfiI, the VHH gene fragments were ligated into SfiI digested and dephosphorylated pCOMB3x (kindly provided by the Scripps Research Institute, La Jolla, CA 92037, USA) using the T4 ligase (Thermo Fisher Scientific, Waltham, MA, USA). Plasmids were cloned into electrocompetent *E. coli* XL1 Blue super competent cells (Agilent, Santa Clara, CA, USA). The bacteria cells were co-infected with M13KO7 helper phages (10^13^ pfu) to generate the naïve VHH phage library according to Barbas et al. [48]. Phage particles were precipitated with ice-cold 20% PEG-8000 in 2.5 M NaCl, incubated for 30 min on ice, and centrifuged for 18 min at 13,000× *g* and 4 °C. Phage particles were resuspended in phosphate-buffered saline with 1% bovine serum albumin (3% BSA/1x PBS (*v*/*w*)) and used directly for panning. 

### 4.4. Panning the Naïve VHH Phage Library

Panning rounds were performed according to [48] with slight modifications. Before each panning round, microtiter plates (Nunc, Rochester, NY, USA) were coated with carrier protein avidin (10 µg/mL) alone and with *Homo sapiens* biotinylated, repetitive extended double-stranded (ds) miRNA-223 (hsa bio ds miRNA-223 XLong, Riboxx GmbH, Dresden, Germany) conjugated on 10 µg/mL avidin. The hsa bio ds miRNA-223 XLong was used as a model ds miRNA construct. The fixation of antigens was performed for 2 h under humid conditions at 37 °C. After blocking steps with 100 µL 3% BSA/1x PBS (*w*/*v*), a pre-panning round was performed. The VHH phage library was pre-incubated against the carrier protein avidin for 30 min at 37 °C. Unbound phage particles were then incubated with decreasing amounts of hsa bio ds miRNA-XLong starting with 100 pmol, 50 pmol to 20 pmol in subsequent panning rounds for 2 h at 37 °C. Bound phage particles were eluted by a pH shift with 100 mM glycine-HCl (pH 2.2) and neutralized with 2 M Tris-HCl, pH 8.0. Eluted phages were used to re-infect the *E. coli* XL1 blue cells (NEB 5-alpha F`I^q^ Competent *E. coli*, Ipswich, MA, USA) according to Barbas et al. for the enrichment of antigen-specific phage particles [48]. After each panning, the output and input titer of phages were determined. The percentage enrichment was calculated by the division of the titer of output phages and titer of input phages multiplied by 100% for each panning round. 

### 4.5. Monoclonal Phage Enzyme-Linked Immunosorbent Assay (ELISA)

After the third panning round, individual colonies were randomly selected to produce monoclonal phage particles as described previously [49]. The obtained monoclonal phage particles were tested for the hsa ds miRNA-223 XLong recognition in a direct ELISA. Additionally, the monoclonal phage particles were tested for cross-reactivity to avidin and the blocking solution. Antigens (10 µg/mL avidin, 20 pmol hsa bio ds miRNA-XLong (Riboxx GmbH, Radebeul, Germany) conjugated on 10 µg/mL avidin and 3% BSA/PBS) were coated on a microtiter plate (Nunc, Rochester, NY, USA) for 2 h at 37 °C. The undiluted monoclonal phage supernatants were incubated for 2 h at 37 °C. Bound phages were detected with horseradish peroxidase (HRP)-conjugated monoclonal mouse anti-M13 antibody (1:7000; MM05H, antibodies-online GmbH, Aachen, Germany). The secondary HRP-labeled anti-M13 antibody was incubated for 1 h at 37 °C. Additionally, a coating control for the immobilized hsa ds miRNA-223 XLong conjugated on avidin was carried out using the commercially available murine anti-DNA:RNA antibody (clone S9.6, MABE 1095, Merck, Darmstadt Deutschland), that was detected with HRP-labeled goat anti-mouse IgG (Fcɣ) antibody respective (1:10,000; 115-035-071, Dianova, Hamburg, Germany). Colorimetric signals were detected after adding tetramethylbenzidine (TMB) peroxidase substrate and 1 M sulfuric acid after 7 min to stop the reaction. The absorbance was measured at 450 nm and 620 nm in an ELISA plate reader.

### 4.6. Expression and Purification of Recombinant VHH_19

The positive clone (pCOMB3x-VHH_19) was first purified with the NucleoSpin^®^ Plasmid Kit (Macherey-Nagel, Dueren, Germany) and sequenced by LGC Genomics GmbH (Berlin, Germany) with M13rev2 sequencing primer provided by the company. The resulting DNA sequence was translated into an amino acid sequence (Kabat numbering scheme) and analyzed for the identification of the four hallmarks indicating a camelid single-domain antibody (VHH). pCOMB3x-VHH_19 was transferred into chemically competent non-suppressor *E. coli* strain HB2151 and expressed as previously described in [44]. The soluble His-tagged VHH_19 was purified from the periplasmic fraction via Ni-NTA affinity chromatography according to the manufacturer’s standard protocol (Protino^®^ Ni-NTA Agarose, Macherey-Nagel, Dueren, Germany).

### 4.7. Generation of Full-Length Recombinant Camelid Heavy-Chain Only Antibody L19

The full-length recombinant camelid heavy chain antibody named L19 was composed of the camelid single-domain antibody (VHH_19) and the *Lama glama* IgG2b constant region (Fc region; GeneBank, accession number AY874455). The DNA sequences encoding for the VHH_19, camelid Fc of IgG2b, GFP, and for the targeting signal ensuring the secretion of expressed antibody into the culture medium were cloned into the expression vector pMC19 using the In-Fusion Cloning Kit following the manufacturer’s protocol (Takara Bio). The expression vector was transfected into the HEK-293 cells using the NeonTM transfection system (Invitrogen, Carlsbad, CA, USA) following the manufacturer’s instructions. The cells were cultivated in Gibco RPMI-1640 medium (Thermo Fisher, Waltham, MA, USA) supplemented with 10% fetal calf serum (FCS) (Thermo Fisher, Waltham, MA, USA), 1% L-glutamine, and 1% beta-mercaptoethanol at 37 °C. Seven days after the transfection, a puromycin-induced selection (2 µg/mL) was performed for two weeks to establish stably producing cell clones. 

### 4.8. ELISA to Detect Secreted L19 Antibodies 

To prove the presence of the secreted camelid heavy-chain-only antibody L19, culture supernatants were tested in a sandwich ELISA. The murine monoclonal anti-camelid IgG2/3 antibody (5 µg/mL; ABIN1981268, antibodies-online, Germany) was coated onto the solid phase of an ELISA microtiter plate for 2 h at 37 °C under humid conditions. After blocking with 5% newborn calf serum (NCS) in 1x PBS for 30 min, undiluted culture supernatants were incubated for 45 min at RT. Moreover, a set of negative controls were considered such as the blocking solution, the pure RPMI medium with supplements, culture supernatants of HEK-293 cells producing a therefore irrelevant membrane-bound protein (MBP), and the culture supernatants of wild-type HEK-293 cells. Next, bound antibodies were detected with 1 µg/mL HRP-conjugated anti-camelid IgG1/2/3 antibodies (murine anti-camelid IgG antibody, ABIN1981270, antibodies-online, Germany) and murine anti-camelid IgG1 antibody (ABIN1981271, antibodies-online, Germany). Detection antibodies were incubated for 45 min at RT. Colorimetric signals were detected after adding tetramethylbenzidine (TMB) peroxidase substrate and 1 M sulfuric acid stop solution after 10 min. The absorbance was measured at 450 nm and 620 nm in an ELISA plate reader. 

### 4.9. Purification of L19 from the Culture Supernatant

Protein A affinity chromatography (ProSep-vA Ultra Chromatography Media, Millipore, Schwalbach, Germany) was used for the purification of L19 from the culture supernatant. For the purification, 500 mL of the collected culture supernatant containing the secreted L19 was mixed 2:1 with the protein A binding buffer (4 M NaCl, 2 M glycine, pH 8.5) and 750 mL were loaded onto the protein A column overnight at 4 °C. The column was washed with a washing buffer (protein A binding buffer diluted 1:3 in ddH2O). The elution of the recombinant heavy chain antibody was performed according to [50]. The purified L19 was dialyzed (Snake Skin^TM^ Dialysis Tubing, cut-off 30 kDa, Thermo Fisher Scientific, Waltham, MA, USA) against 1x PBS overnight at 4 °C. The concentration of dialyzed L19 was determined by using the BCA protein assay method according to the manufacturer’s instructions (Pierce^TM^ BCA Protein Assay Kit, Thermo Fisher Scientific, Waltham, MA, USA). L19 was stored at 4 °C. 

### 4.10. Sodium Dodecyl Sulfate Polyacrylamide Gel Electrophoresis (SDS-PAGE) and Western Blot Analysis

Samples collected during the Ni-NTA purification of His-tagged VHH_19, the full-length antibody L19, and a murine monoclonal antibody were analyzed via SDS-PAGE and WB. VHH_19 samples were separated on a 12% precast Mini-PROTEAN TGX gel (BioRad, München, Germany). Camelid L19 (2 µg) and the murine antibody were separated on 10% SDS polyacrylamide gel under reducing conditions using a buffer containing ß-mercaptoethanol (ROTI^®^Load 1; Carl Roth GmbH, Karlsruhe, Germany). All samples were prepared according to the manufacturer’s instructions. Next, the protein bands were electrophoretically transferred to a nitrocellulose membrane (Trans-Blot TurboTM mini-size nitrocellulose, 0.2 µm, BioRad, München, Germany) under semi-dry conditions (2.6 A, 7 min) with the Trans-Blot Turbo Transfer System (BioRad, München, Germany). To reduce non-specific binding, a blocking step was performed with 1x Tris-buffered saline/0.05% Tween20 (*v*/*v*) (TBS-T)/5% goat serum (*v/v*) (Biowest, Riverside, CA, USA) for 30 min at RT. For the detection of HA-tagged VHH_19, the membrane was incubated with a mouse anti-HA-tag antibody (1:5000; 16B12, BioLegend, San Diego, CA, USA) overnight at 4 °C, washed three times with 1x TBS-T, and incubated with the secondary antibody (1:10,000; HRP-conjugated goat-anti-mouse IgG (Fcɣ) antibody, 115-035-071, Dianova, Hamburg, Germany) for 1 h at RT. For detection of camelid L19 and murine antibodies, the membranes were incubated with HRP-conjugated murine anti-camelid IgG2/3 antibody (1:7000; ABIN1981272, antibodies-online, Germany), murine anti-camelid IgG1 antibody (1:7000; ABIN1981271, antibodies-online, Germany) and HRP-conjugated goat anti-mouse IgG (H + L) antibody respectively (1:10,000; 0300-0108P, BioRad, München, Germany) overnight at 4 °C. After three washing steps with TBS-T, protein bands were visualized by the substrate solution (ClarityTM Western ECL Substrate, BioRad, München, Germany) on ChemiDoc MP Imaging Systems (BioRad, München, Germany).

### 4.11. ELISA with the Purified L19

L19 (25 µg/mL) was coated onto the solid phase of a microtiter ELISA plate for 2 h at 37 °C under humid conditions. After blocking with 1x PBS/5% NCS (*v*/*v*) for 30 min at RT, hsa bio ds miRNA-223, -197, -379, -30, and -let7f XLong (10 µM, 5 µM, 2.5 µM, 1 µM) were incubated for 45 min at RT. The blocking solution served as the negative control and was added instead of any miRNA probe. Next, the bound biotinylated ds miRNAs were incubated with SAV-HRP (1:10,000; Roche, Mannheim, Germany) for 45 min at RT. Colorimetric signals were induced by adding tetramethylbenzidine (TMB) peroxidase substrate. The reaction was stopped after 15 min with 1 M sulfuric acid. The absorbance was measured at 450 nm and 620 nm in an ELISA plate reader.

### 4.12. Software

Statistical analyses were performed using GraphPad Prism (version 8.4.1). The primer modifications were generated with SnapGene (version 4.2.6). Chemiluminescence signals of protein bands were analyzed with Image Lab Software (version 2.0, BioRad, München, Germany). 

## Figures and Tables

**Table 1:** Primer sequences for the amplification of VHH DNA sequences.

Primer Name	Sequence (5′ → 3′)	Reference
P12	GTCCTGGCTGCTCTTCTACAAGG	[46]
P13	ATGGAGAGGACGTCCTTGGGT	[46]
mL93	ACCGTGGCCCAGGCGGCCCAGGTGCAGCTGCAGGAGTCTGGRGGAGG	[47] modified
mL94	GTGCTGGCCGGCCTGGCCGCTGGAGACGGTGACCTGGGT	[47] modified
mL95	GTGCTGGCCGGCCTGGCCTGAGGAGACGGTGACCTGGGT	[47] modified

**Figure A1:**
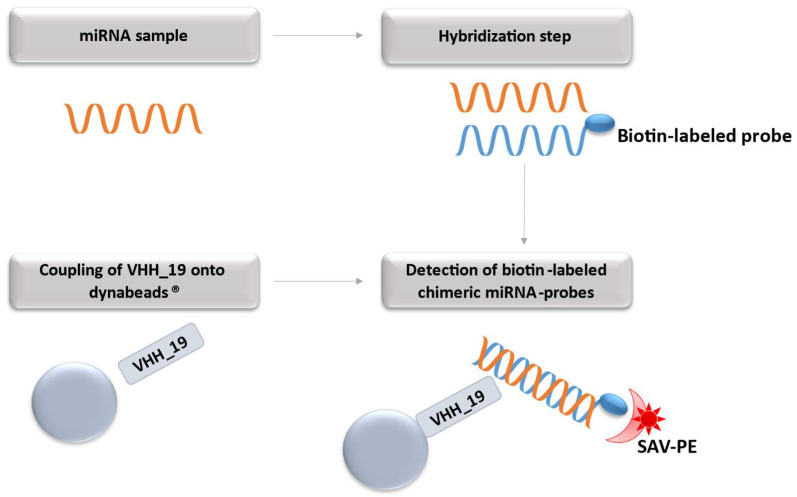
Schematic overview of the detection of miRNA with the anti-nucleic binder VHH_19. The miRNA sample (synthetic miRNA or isolated from human serum) was hybridized with the biotin-labeled complementary probe. The complex of biotin-labeled chimeric miRNA-probes was incubated with VHH_19 coupled dynabeads^®^. The detection of bound miRNA probes to VHH_19 occurred by the binding of Phycoerythrin-labeled SAV (SAV-PE) to the biotin.

## Data Availability

The data presented in this study are available on request from the corresponding author. The data are not publicly available.

## Appendix A

For this assay (Table A1) the used miRNAs were synthetically generated and hybridized to the complementary sequences and detected with SAV-PE (Figure A1). The miRNA-93 was detected at the concentration of 0.1 fmol/µL. The weak signal may correlate to the very low concentration used for this assay. Moreover, the no signal for miRNA-223 may be explained by the phenomenon of loop formation during the hybridization step caused by the imperfect matching of two single-stranded miRNAs. Nevertheless, further assays have to be established to prove the suitability of VHH_19 for bead-based miRNA detection.

**Table A1:** Detection of synthetic miRNA with VHH_19 via bead-based assay. FI stands for fluorescence intensity.

Concentration of Synthetic RNA		hsa bio ds miRNA-93 XLong	hsa bio ds miRNA-223 XLong
		FI	FI
0.1 fmol/µL	without VHH_19	17	2
0.01 fmol/µL		0	0
1 atom/L		0	0
0.1 fmol/µL	with VHH_19	137	3
0.01 fmol/µL		0	0
1 atom/L		0	0

For the assay presented in Table A2, miRNA from healthy and diseased donor samples was isolated according to the manufacturer’s protocol (mirVANA^TM^ PARIS^TM^ RNA and the Native Protein Purification Kit, Thermo Fisher Scientific, Waltham, MA, USA) and hybridized with biotinylated miRNA-93 and miRNA-223 complementary capture sequences. The miRNA-capture probe hybrids were incubated with VHH_19 coupled dynabeads and bound complexes were detected by SAV-PE. The signal for the miRNA-93 was higher in the sample from diseased patients compared to the ones from healthy donors which might be due to the overexpression of this miRNA during DCM. In the case of miRNA-223, the signal is higher in the healthy patient sample indicating that the miRNA-223 might be underexpressed in the association with DCM.

**Table A2:** Detection of miRNA isolated from human serum with VHH_19 via bead-based assay. FI stands for fluorescence intensity.

miRNA Real Sample		miRNA-93	miRNA-223
		FI	FI
Diseased	Without VHH_19	20	10
	with VHH_19	+ (16)	− (0)
Healthy	Without VHH_19	11	11
	with VHH_19	− (0)	+ (10)

## References

[1] Wienholds E., Plasterk R.H.A. (2005). MicroRNA Function in Animal Development. FEBS Lett..

[2] Cressatti M., Juwara L., Galindez J.M., Velly A.M., Nkurunziza E.S., Marier S., Canie O., Gornistky M., Schipper H.M. (2020). Salivary MicroR-153 and MicroR-223 Levels as Potential Diagnostic Biomarkers of Idiopathic Parkinson’s Disease. Mov. Disord..

[3] Ding H., Wang Y., Hu L., Xue S., Wang Y., Zhang L., Zhang Y., Qi H., Yu H., Aung L.H.H. (2020). Combined Detection of MiR-21-5p, MiR-30a-3p, MiR-30a-5p, MiR-155-5p, MiR-216a and MiR-217 for Screening of Early Heart Failure Diseases. Biosci. Rep..

[4] Fauth M., Hegewald A.B., Schmitz L., Krone D.J., Saul M.J. (2019). Validation of Extracellular MiRNA Quantification in Blood Samples Using RT-qPCR. FASEB BioAdvances.

[5] Martinez B., Peplow P. (2020). MicroRNAs in Blood and Cerebrospinal Fluid as Diagnostic Biomarkers of Multiple Sclerosis and to Monitor Disease Progression. Neural. Regen. Res..

[6] Raeisi F., Mahmoudi E., Dehghani-Samani M., Hosseini S.S.E., Ghahfarrokhi A.M., Arshi A., Forghanparast K., Ghazanfari S. (2020). Differential Expression Profile of MiR-27b, MiR-29a, and MiR-155 in Chronic Lymphocytic Leukemia and Breast Cancer Patients. Mol. Ther.-Oncolytics.

[7] Siegismund C.S., Rohde M., Kühl U., Escher F., Schultheiss H.P., Lassner D. (2016). Absent MicroRNAs in Different Tissues of Patients with Acquired Cardiomyopathy. Genom. Proteom. Bioinform..

[8] Wen Y., Han J., Chen J., Dong J., Xia Y., Liu J., Jiang Y., Dai J., Lu J., Jin G. (2015). Plasma MiRNAs as Early Biomarkers for Detecting Hepatocellular Carcinoma: Plasma MiRNAs and Hepatocellular Carcinoma. Int. J. Cancer.

[9] Zajdel M., Rymkiewicz G., Sromek M., Cieslikowska M., Swoboda P., Kulinczak M., Goryca K., Bystydzienski Z., Blachnio K., Ostrowska B. (2019). Tumor and Cerebrospinal Fluid MicroRNAs in Primary Central Nervous System Lymphomas. Cancers.

[10] Aleshcheva G., Pietsch H., Escher F., Schultheiss H. (2021). MicroRNA Profiling as a Novel Diagnostic Tool for Identification of Patients with Inflammatory and/or Virally Induced Cardiomyopathies. ESC Heart Fail..

[11] Winfield J.B., Faiferman I., Koffler D. (1977). Avidity of Anti-DNA Antibodies in Serum and IgG Glomerular Eluates from Patients with Systemic Lupus Erythematosus. Association of High Avidity Antinative DNA Antibody with Glomerulonephritis. J. Clin. Investig..

[12] Bizzaro N., Villalta D., Giavarina D., Tozzoli R. (2012). Are Anti-Nucleosome Antibodies a Better Diagnostic Marker than Anti-DsDNA Antibodies for Systemic Lupus Erythematosus? A Systematic Review and a Study of Metanalysis. Autoimmun. Rev..

[13] Gualtierotti R., Biggioggero M., Penatti A.E., Meroni P.L. (2010). Updating on the Pathogenesis of Systemic Lupus Erythematosus. Autoimmun. Rev..

[14] Mehra S., Fritzler M.J. (2014). The Spectrum of Anti-Chromatin/Nucleosome Autoantibodies: Independent and Interdependent Biomarkers of Disease. J. Immunol. Res..

[15] Stollar B.D. (1970). Double-Helical Polynucleotides: Immunochemical Recognition of Differing Conformations. Science.

[16] Cros P., Kurfiirst R., Allibert P., Battail N., Piga N., Roig V., Thuong N.T., Mandrand B., Hélène C. (1994). Monoclonal Antibodies Targeted to A-Oligonucleotides. Characterisation and Application in Nucleic Acid Detection. Nucleic Acids Res..

[17] Nakazato H. (1979). Radioimmunoassay of an Antibody to ΦX174 DNA·RNA Hybrid. Anal. Biochem..

[18] Kitagawa Y., Matsumoto T., Okuhara E., Shikata E. (1977). Immunogenicity of Rice Dwarf Virus-Ribonucleic Acid. Tohoku J. Exp. Med..

[19] Ikegami M., Francki R.I.B. (1973). Presence of Antibodies to Double-Stranded RNA in Sera of Rabbits Immunized with Rice Dwarf and Maize Rough Dwarf Viruses. Virology.

[20] Hu Z., Leppla S.H., Li B., Elkins C.A. (2014). Antibodies Specific for Nucleic Acids and Applications in Genomic Detection and Clinical Diagnostics. Expert Rev. Mol. Diagn..

[21] Stollar B.D., Voss E.W. (1986). Antibodies to DN. Crit. Rev. Biochem..

[22] David Stollar B. (1980). The Experimental Induction of Antibodies to Nucleic Acids. Methods in Enzymology.

[23] Erlanger B.F., Beiser S.M. (1964). Antibodies specific for ribonucleosides and ribonucleotides and their reaction with dna. Proc. Natl. Acad. Sci. USA.

[24] Boguslawski S.J., Smith D.E., Michalak M.A., Mickelson K.E., Yehle C.O., Patterson W.L., Carrico R.J. (1986). Characterization of Monoclonal Antibody to DNA · RNA and Its Application to Immunodetection of Hybrids. J. Immunol. Methods.

[25] De Meyer T., Muyldermans S., Depicker A. (2014). Nanobody-Based Products as Research and Diagnostic Tools. Trends Biotechnol..

[26] Van der Linden R.H.J., Frenken L.G.J., de Geus B., Harmsen M.M., Ruuls R.C., Stok W., de Ron L., Wilson S., Davis P., Verrips C.T. (1999). Comparison of Physical Chemical Properties of Llama VHH Antibody Fragments and Mouse Monoclonal Antibodies. Biochim. Et Biophys. Acta (BBA)—Protein Struct. Mol. Enzymol..

[27] Conrath K., Vincke C., Stijlemans B., Schymkowitz J., Decanniere K., Wyns L., Muyldermans S., Loris R. (2005). Antigen Binding and Solubility Effects upon the Veneering of a Camel VHH in Framework-2 to Mimic a VH. J. Mol. Biol..

[28] Dumoulin M., Conrath K., Van Meirhaeghe A., Meersman F., Heremans K., Frenken L.G.J., Muyldermans S., Wyns L., Matagne A. (2009). Single-Domain Antibody Fragments with High Conformational Stability. Protein Sci..

[29] Muyldermans S. (2013). Nanobodies: Natural Single-Domain Antibodies. Annu. Rev. Biochem..

[30] Kabat E.A., Wu T.T., Perry H.M., Gottesman K.S., Foeller C. (1991). Sequences of Proteins of Immunological Interest.

[31] Benes V., Castoldi M. (2010). Expression Profiling of MicroRNA Using Real-Time Quantitative PCR, How to Use It and What Is Available. Methods.

[32] Creighton C.J., Reid J.G., Gunaratne P.H. (2009). Expression Profiling of MicroRNAs by Deep Sequencing. Brief. Bioinform..

[33] Davison T.S., Johnson C.D., Andruss B.F. (2006). Analyzing Micro-RNA Expression Using Microarrays. Methods in Enzymology.

[34] Ponsel D., Neugebauer J., Ladetzki-Baehs K., Tissot K. (2011). High Affinity, Developability and Functional Size: The Holy Grail of Combinatorial Antibody Library Generation. Molecules.

[35] Sabir J.S.M., Atef A., El-Domyati F.M., Edris S., Hajrah N., Alzohairy A.M., Bahieldin A. (2014). Construction of Naïve Camelids VHH Repertoire in Phage Display-Based Library. Comptes Rendus Biol..

[36] Saerens D., Frederix F., Reekmans G., Conrath K., Jans K., Brys L., Huang L., Bosmans E., Maes G., Borghs G. (2005). Engineering Camel Single-Domain Antibodies and Immobilization Chemistry for Human Prostate-Specific Antigen Sensing. Anal. Chem..

[37] Harmsen M.M., Fijten H.P.D. (2012). Improved functional immobilization of llama single-domain antibody fragments to polystyrene surfaces using small peptides. J. Immunoass. Immunochem..

[38] Orenes-Piñero E., Marín F., Lip G.Y.H. (2016). MiRNA-197 and MiRNA-223 and Cardiovascular Death in Coronary Artery Disease Patients. Ann. Transl. Med..

[39] Geithe C., Zeng B., Schmidt C., Dinter F., Roggenbuck D., Lehmann W., Dame G., Schierack P., Hanack K., Rödiger S. (2021). A Multiplex Microchamber Diffusion Assay for the Antibody-Based Detection of MicroRNAs on Randomly Ordered Microbeads. Biochemistry.

[40] Kappel A., Backes C., Huang Y., Zafari S., Leidinger P., Meder B., Schwarz H., Gumbrecht W., Meese E., Staehler C.F. (2015). MicroRNA In Vitro Diagnostics Using Immunoassay Analyzers. Clin. Chem..

[41] Tran H.V., Piro B., Reisberg S., Duc H.T., Pham M.C. (2013). Antibodies Directed to RNA/DNA Hybrids: An Electrochemical Immunosensor for MicroRNAs Detection Using Graphene-Composite Electrodes. Anal. Chem..

[42] Ye J.-D., Tereshko V., Frederiksen J.K., Koide A., Fellouse F.A., Sidhu S.S., Koide S., Kossiakoff A.A., Piccirilli J.A. (2008). Synthetic Antibodies for Specific Recognition and Crystallization of Structured RNA. Proc. Natl. Acad. Sci. USA.

[43] Jung E., Lee J., Hong H.J., Park I., Lee Y. (2014). RNA Recognition by a Human Antibody against Brain Cytoplasmic 200 RNA. RNA.

[44] Schlör A., Holzlöhner P., Listek M., Grieß C., Butze M., Micheel B., Hentschel C., Sowa M., Roggenbuck D., Schierack P. (2018). Generation and Validation of Murine Monoclonal and Camelid Recombinant Single Domain Antibodies Specific for Human Pancreatic Glycoprotein 2. New Biotechnol..

[45] Zola H., Fusco M., Macardle P.J., Flego L., Roberton D. (1995). Expression of Cytokine Receptors by Human Cord Blood Lymphocytes: Comparison with Adult Blood Lymphocytes. Pediatr. Res..

[46] Dong J., Thompson A.A., Fan Y., Lou J., Conrad F., Ho M., Pires-Alves M., Wilson B.A., Stevens R.C., Marks J.D. (2010). A Single-Domain Llama Antibody Potently Inhibits the Enzymatic Activity of Botulinum Neurotoxin by Binding to the Non-Catalytic α-Exosite Binding Region. J. Mol. Biol..

[47] Pardon E., Laeremans T., Triest S., Rasmussen S.G.F., Wohlkönig A., Ruf A., Muyldermans S., Hol W.G.J., Kobilka B.K., Steyaert J. (2014). A General Protocol for the Generation of Nanobodies for Structural Biology. Nat. Protoc..

[48] (2004). Phage Display; Cold Spring Harbor Laboratory Pr: 2004.

[49] Coomber D.W.J. (2001). Panning of Antibody Phage-Display Libraries: Standard Protocols. Antibody Phage Display.

[50] Holzlöhner P., Butze M., Maier N., Hebel N., Schliebs E., Micheel B., Füner J., Heidicke G., Hanack K. (2018). Generation of Murine Monoclonal Antibodies with Specificity against Conventional Camelid IgG1 and Heavy-Chain Only IgG2/3. Vet. Immunol. Immunopathol..

